# Biocompatibility and Bioactivity of Set Direct Pulp Capping Materials on Human Dental Pulp Stem Cells

**DOI:** 10.3390/ma13183925

**Published:** 2020-09-04

**Authors:** Yemi Kim, Donghee Lee, Dani Song, Hye-Min Kim, Sin-Young Kim

**Affiliations:** 1 Department of Conservative Dentistry, Dental Research Institute, Ewha Womans University School of Medicine, Seoul 07985, Korea; yemis@hanmail.net; 2 College of Medicine, The Catholic University of Korea, Seoul 06591, Korea; dong524@naver.com; 3 Department of Conservative Dentistry, Seoul St. Mary’s Hospital, College of Medicine, The Catholic University of Korea, 222 Banpo-daero, Seocho-gu, Seoul 06591, Korea; dksl0104@gmail.com (D.S.); hmtoto@naver.com (H.-M.K.)

**Keywords:** cell viability, cell migration, pulp capping materials, ALP activity, ARS assay

## Abstract

In this study, we assessed the biocompatibility and bioactivity of various pulp capping materials—ProRoot MTA (Dentsply Tulsa Dental Specialties), Biodentine (Septodont), TheraCal LC (Bisco), and Dycal (Dentsply Caulk)—on human dental pulp stem cells (hDPSCs). Experimental disks (diameter, 7 mm; height, 4 mm) were stored in a humified incubator at 37 °C for 48 h. Then, the pulp capping materials were tested for cytotoxic effects by methyl-thiazoldiphenyl-tetrazolium and scratch wound healing assays, and for mineralization potential by Alizarin red S (ARS) staining assay and alkaline phosphatase enzyme (ALP) activity. Cell viability and cell migration did not significantly differ between ProRoot MTA, Biodentine, and control (*p* > 0.05). TheraCal LC exhibited slower cell migration on days 2–4 compared to control (*p <* 0.05), and Dycal showed no cell migration. ALP activity was highest with Biodentine on days 10 and 14, and was lowered with TheraCal LC and Dycal (*p* < 0.05). In the ARS assay, hDPSCs grown in ProRoot MTA and TheraCal LC eluates showed significantly increased mineralized nodule formation on day 21 compared to Biodentine, Dycal, and control (*p* < 0.05). These findings indicate that ProRoot MTA, Biodentine, and TheraCal LC exhibit better biocompatibility and bioactivity than Dycal.

## 1. Introduction

Direct pulp capping procedures are usually performed to replace the dentin-pulp complex and maintain pulp vitality in cases of pin-point pulp exposure after caries removal or complicated crown fracture [1,2]. The conventional pulp capping material is calcium hydroxide, which is considered the gold standard of direct pulp capping [3]. Calcium hydroxide has a high pH of 12 and releases Ca^2+^ and OH^−^. Placement of calcium hydroxide on exposed pulp leads to the formation of a superficial necrotic zone, followed by the stimulation of the mineralization process directly against this necrotic zone [3]. However, the calcium hydroxide product Dycal (Dentsply Caulk, Midford, DE, USA) has several disadvantages, including high solubility, stimulation of coagulative necrosis and inflammatory processes, and formation of tunnel defects with subsequent bacterial invasion [4].

Mineral trioxide aggregate (MTA) was developed due to its tight sealing potential and favorable biological properties; it also has odontoblastic and cementoblastic properties [5,6,7]. Previous studies have demonstrated favorable clinical outcomes following direct pulp capping with MTA compared to calcium hydroxide [8,9,10,11]. A clinical study reported a 42-month success rate of nearly 80% with MTA compared to 59% with calcium hydroxide [9]. However, MTA has several disadvantages, including its extended setting time, properties that make handling difficult, and discoloration. The drawbacks of both MTA and calcium hydroxide led to the development of calcium silicate-based cements. Biodentine (Septodont, Saint-Maur-des-Fossés, France) exhibits fast setting, no discoloration, and similar clinical results compared to ProRoot MTA (Dentsply Tulsa Dental Specialties, Tulsa, OK, USA) [12,13,14], and is thus considered a suitable pulp capping material to replace MTA.

Recently, resin-modified calcium silicates have been introduced, which are enhanced materials that exhibit several advantages, including higher mechanical strength and the ability to be immediately light cured, easily manipulated, and precisely applied into the exposed pulp [15]. Resin-modified calcium silicates contain a hydrophilic monomer and release higher levels of Ca^2+^ and OH^−^ compared to ProRoot MTA and Dycal [16], thereby providing antibacterial action and stimulating dentin formation. The resin-modified calcium silicate product TheraCal LC (Bisco, Schamberg, IL, USA) has a fluid-like consistency prior to light curing, facilitating easy application. Furthermore, an upper restorative procedure can be immediately performed to completely seal the tooth. However, TheraCal LC reportedly shows more cytotoxic properties to human dental pulp stem cells (hDPSCs) compared to ProRoot MTA and Biodentine [17,18].

In this study, we aimed to compare various calcium silicate-based pulp capping materials with conventional calcium hydroxide in terms of biocompatibility and bioactivity on hDPSCs.

## 2. Materials and Methods

### 2.1. Culture of Human Dental Pulp Stem Cells (hDPSCs)

The hDPSC lines were obtained and grown by a previously reported method [19]. The cells were placed at 37 °C in a 100% humidity chamber with 5% CO_2_. A colony-forming test showed that most of the hDPSCs exhibited a spindle form morphology, which is in accordance with other types of mesenchymal stem cells. This study protocol was approved by the institutional review board of Seoul St. Mary’s Hospital, College of Medicine, the Catholic University of Korea (IRB No. KC19SNSI0186).

### 2.2. Production of Experimental Pulp Capping Material Disks

The investigated direct pulp capping materials were ProRoot MTA (Dentsply Tulsa Dental Specialties), Biodentine (Septodont), TheraCal LC (Bisco), and Dycal (Dentsply Tulsa Dental Specialties) (Table 1). All experimental materials were prepared following the manufacturer’s recommendations. From each material, we created disks, with a diameter of 7 mm and height of 4 mm, using a previously reported method [20].

### 2.3. Evaluation of Cell Viability

Cell viability was assessed by a methyl-thiazoldiphenyl-tetrazolium (MTT) assay (MTT Cell Growth Assay Kit, Chemicon, Rosemont, IL, USA) [19,21,22]. We seeded hDPSCs at a density of 1.0 × 10^4^ cells/well on 24-well cell culture plates (SPL Life Sciences, Pocheon, Korea). The material disks were placed into inserts with a 0.4 μm pore size (SPLInsert; SPL Life Sciences), and these inserts were maintained over the attached hDPSCs for 5 days. Then, the hDPSCs were treated with MTT solution according to a previously reported method at 0, 1, 2, 3, and 5 days [19]. We measured the absorbance at 570 nm. Positive controls were hDPSCs cultured without experimental disks. Each group was evaluated in quadruplicate.

### 2.4. Evaluation of Cell Migration

Cell migration was assessed by scratch wound healing assay according to a previously reported method [19]. The material disks were placed into inserts with a 0.4 μm pore size (SPLInsert; SPL Life Sciences), and these inserts were maintained over the attached hDPSCs for 4 days. Images of the scratch healing of all groups were acquired at 0, 1, 2, 3, and 4 days with a phase-contrast microscope (Olympus, Tokyo, Japan). Cell migration area was calculated using ImageJ 1.46r (National Institutes of Health, Bethesda, MD, USA). Each group was evaluated in quadruplicate.

### 2.5. Determination of Alkaline Phosphatase Enzyme (ALP) Activity

The experimental disks were eluted in an osteogenic medium at a concentration of 5 mg/mL and maintained in a humidified chamber for 7 days according to a previously reported method [20]. We evaluated the hDPSCs’ ALP activity in the pulp capping material eluate using the Senso-Lyte^®®^ p-nitrophenylphosphate (pNPP) alkaline phosphatase assay kit (AnaSpec, Fremont, CA, USA) following the manufacturer’s method. We seeded hDPSCs at a density of 2.0 × 10^4^ cells/well on 24-well plates (SPL Life Sciences). These cells were grown for 14 days, and then lysed with TritonX-100 (AnaSpec, Fremont, CA, USA). Next, pNPP was added to the supernatant of the cell lysates, and incubated for 30 min at 4 °C. We measured the absorbance at 405 nm. Each group was evaluated in quadruplicate.

### 2.6. Determination of Alizarin Red S (ARS) Staining Assay

Calcified nodule formation among hDPSCs was assessed using an ARS assay [21,22]. We seeded hDPSCs at a density of 2.0 × 10^4^ cells/well on 24-well plates (SPL Life Sciences), and cultured these cells in pulp capping material eluate for 21 days. Next, the hDPSCs were stained with ARS solution according to a previously reported method [20], and we measured the absorbance at 560 nm. Each group was evaluated in quadruplicate.

### 2.7. Alizarin Red S (ARS) Staining Assay of Various Biodentine Eluates

Three different methods were used for ARS assay of the Biodentine group. Two methods involved different preparation of Biodentine disks, with Biodentine powder mixed either with the supplied liquid including calcium chloride (CaCl_2_) or with distilled water (ddH_2_O). In both methods, these disks were eluted in an osteogenic medium at a concentration of 5 mg/mL and maintained in a humidified incubator at 37 °C for 7 days. In the third method, the Biodentine powder was mixed with an osteogenic medium, and this mixture was maintained in a humidified chamber at 37 °C for 7 days. Subsequently, we seeded hDPSCs at a density of 2.0 × 10^4^ cells/well on 24-well plates (SPL Life Sciences) and cultured them over a period of 28 days in the various Biodentine eluates according to the test method. The ARS assay was performed as described above.

### 2.8. Statistical Analyses

We performed statistical analyses with SPSS (ver. 24.0; IBM Corp., Armonk, NY, USA). The Shapiro–Wilk test of normality was used to confirm the distribution of data. Since normal distribution of the data was verified, we used repeated measures analyses of variance for overall comparisons. One-way analysis of variance and Tukey’s post-hoc tests were used for multiple comparisons among experimental groups at each time-point. A *p*-value of <0.05 was considered significant.

## 3. Results

**Figure 1:**
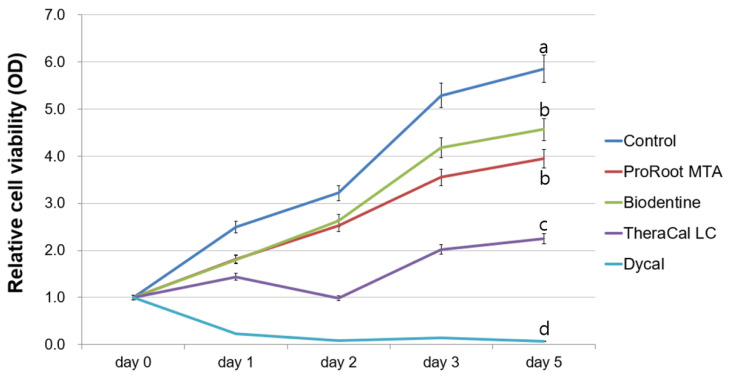
Evaluation of cell viability by methyl-thiazoldiphenyl-tetrazolium (MTT) assay. Different letters indicate statistically significant differences among experimental groups.

The MTT assay revealed that cell viability on days 2–5 in the TheraCal LC group significantly differed from that in the ProRoot MTA and Biodentine groups (*p* < 0.05). Among all experimental groups, the Dycal group exhibited the lowest cell viability after day 1 (*p* < 0.05) (Figure 1).

**Figure 2:**
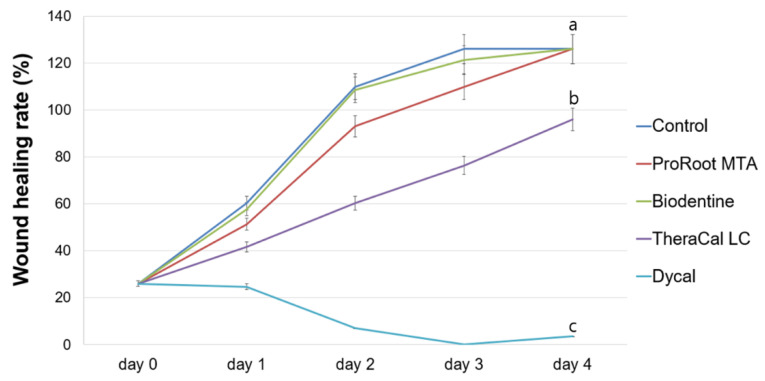
Evaluation of cell migration by wound healing assay. Different letters represent statistically significant differences among experimental groups.

**Figure 3:**
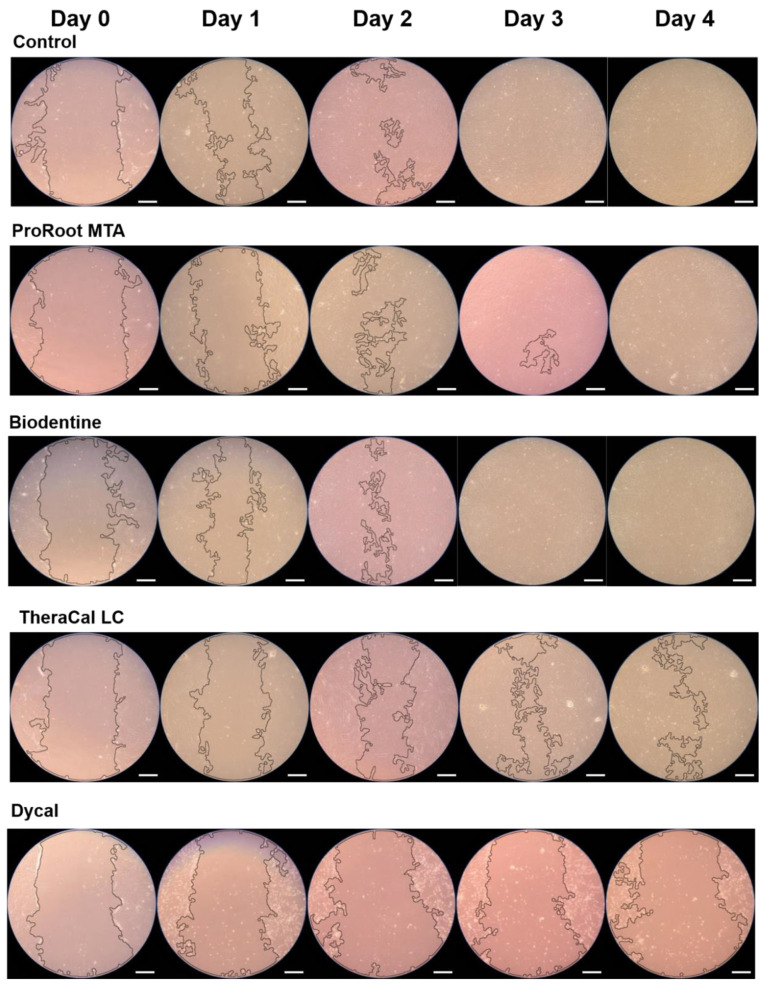
Representative images of scratch wound healing assay (scale bar = 250 μm).

The wound healing assay demonstrated no significant differences between the ProRoot MTA and Biodentine groups during any experimental period (*p* > 0.05). The TheraCal LC group exhibited a slower migration rate on days 2–4 compared to the ProRoot MTA and Biodentine groups (*p <* 0.05). The Dycal group showed no wound healing, and exhibited significant differences compared to the control group on days 1–4 (*p <* 0.05; Figure 2). Representative images are shown in Figure 3.

**Figure 4:**
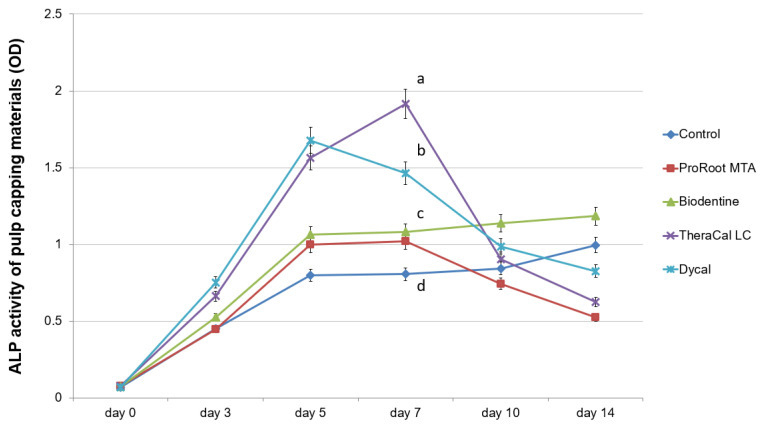
Determination of alkaline phosphatase enzyme (ALP) activity of various pulp capping materials.

ALP enzyme activity over 7 days was significantly higher in the TheraCal LC and Dycal groups compared to the ProRoot MTA and Biodentine groups (*p* < 0.05; Figure 4). ALP activity on days 10 and 14 was highest in the Biodentine group, and decreased in the TheraCal LC and Dycal groups (Figure 4).

**Figure 5:**
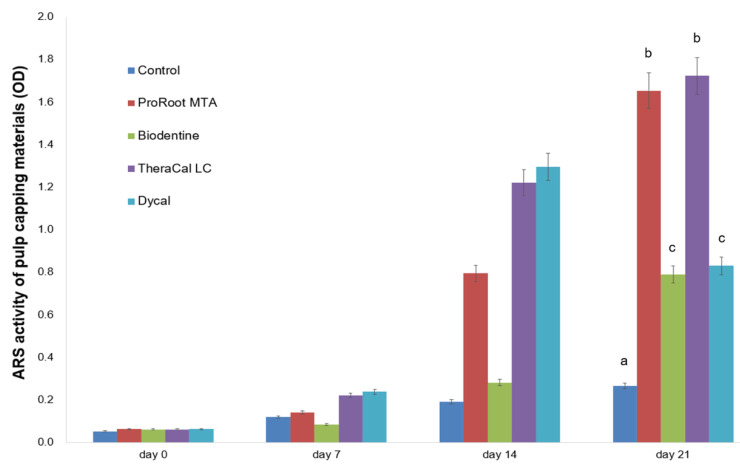
Determination of calcium nodule formation by Alizarin red S (ARS) staining assay. Different letters represent statistically significant differences among experimental groups.

In the ARS assay, mineralized nodule formation on day 21 significantly differed in hDPSCs grown in ProRoot MTA and TheraCal LC eluates compared to those grown in the Biodentine, Dycal, and control groups (*p* < 0.05, Figure 5).

**Figure 6:**
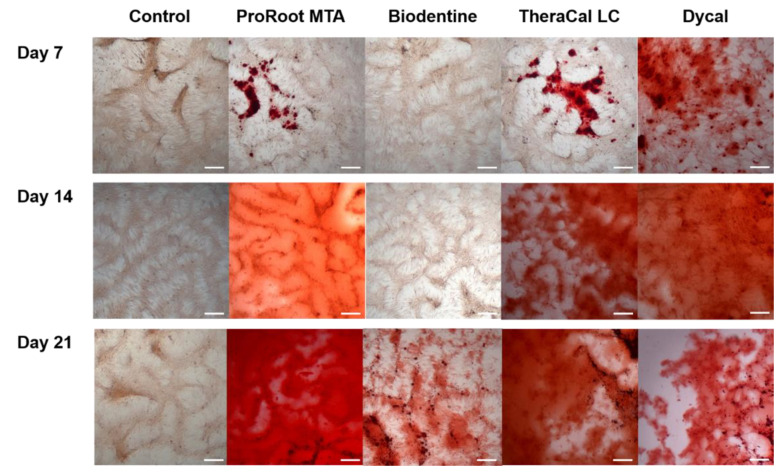
Representative images of Alizarin red S (ARS) staining assay (scale bar = 500 μm).

**Figure 7:**
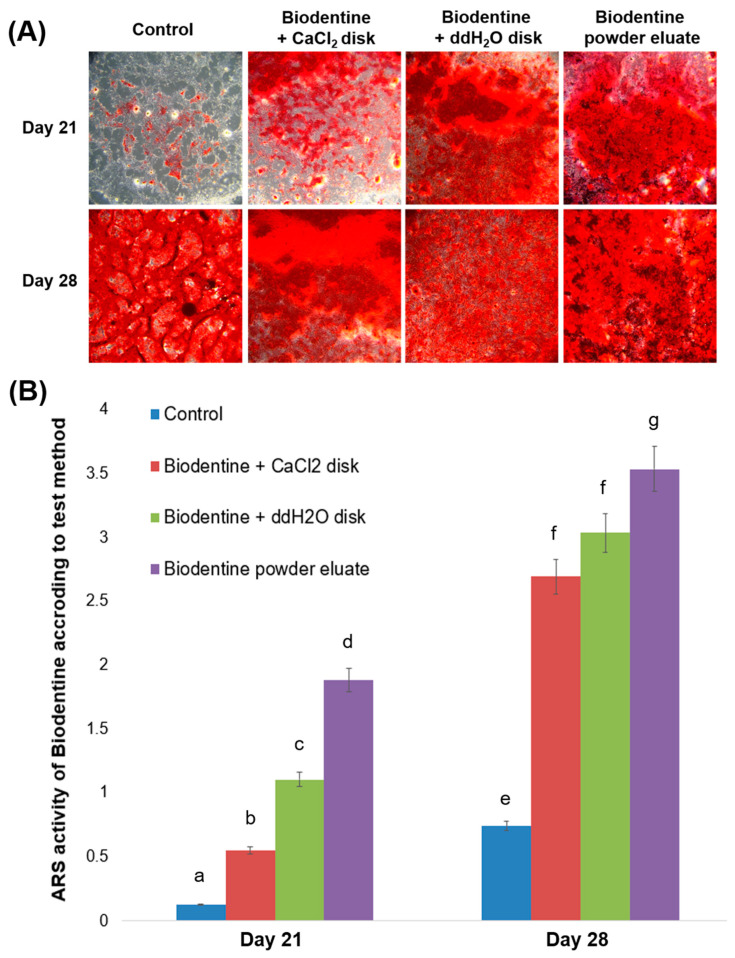
Calcium nodule formation according to Alizarin red S (ARS) staining assay of various Biodentine eluates according to test method. (**A**) Representative images of ARS staining assay, (**B**) ARS activity of various Biodentine eluates. Different superscript letters represent statistically significant differences.

Representative images are shown in Figure 6. Between the different Biodentine methods, calcium nodule formation on day 21 was greatest in the Biodentine powder eluate, followed by the Biodentine disk mixed with ddH_2_O, followed by the Biodentine disk mixed with the supplied liquid including CaCl_2_ (Figure 7, *p* < 0.05). On day 28, we observed no significant difference between the Biodentine disks mixed with ddH_2_O versus CaCl_2_ (Figure 7, *p* > 0.05).

## 4. Discussion

Direct pulp capping procedures are necessary for treating pulpal injuries and stimulating tertiary dentine formation. Materials for direct pulp capping must be noncytotoxic to dental pulp stem cells, and bioactive for inducing odontogenic differentiation. Furthermore, materials that set quickly enable single-visit restoration. In this study, we compared various calcium silicate-based cements with conventional calcium hydroxide in terms of their biocompatibility and bioactivity on hDPSCs.

MTT and wound healing assays were performed to evaluate the biologic properties of ProRoot MTA, Biodentine, TheraCal LC, and Dycal. Compared to the Dycal group, the ProRoot MTA, Biodentine, and control groups all showed superior cell viability and migration ability. The cell viability and cell migration rate in the TheraCal LC group were lower than in the control group, but higher than in the Dycal group. In the Dycal group, the cells were dead after 24 h, and scratch wounds did not heal at all.

Biodentine is an innovative bioceramic calcium-silicate-based cement that exhibits noncytotoxic properties and good pulpal responses [20,23,24,25]. When used for pulp capping procedures, Biodentine can preserve pulp vitality and induce tertiary dentin formation [25]. De Rossi et al. used Biodentine after pulpotomy in dogs’ teeth, and reported that Biodentine exhibits tissue biocompatibility and forms a greater thickness of dentinal bridge compared to MTA [25]. Biodentine has greater color stability compared to ProRoot MTA [26], and is thus a popular choice for areas with aesthetic concerns.

In a prior study of various calcium silicate materials used for direct pulp capping, Biodentine was associated with the highest migration rate of hDPSCs, and scanning electron microscopy revealed higher cell adhesion on the Biodentine disk [23]. The authors further reported that Biodentine lacked Sr, Al, and S, which are associated with cytotoxic properties of other direct pulp capping materials [23]. Our present results showed that Biodentine and ProRoot MTA are both noncytotoxic to hDPSCs. Compared to the Biodentine and ProRoot MTA groups, the TheraCal LC group showed substantially reduced cell viability. TheraCal LC contains a hydrophilic resin monomer, hydrophobic resin monomer, and hydrophilic filler in addition to Portland cement powder [27]. Methacrylate resin components adversely influence cell membranes through alteration of the lipid bilayer. Previous studies reported reduced cell metabolism and incomplete tissue bridges after contact of hDPSCs with TheraCal LC cement [18,28], and cells cultured in medium containing TheraCal LC show significantly increased IL-8 levels [29]. In our present study, the Dycal group exhibited significant cell toxicity compared to other groups, which is in accordance with previous studies [30,31].

In this study, we analyzed the mineralization ability of ProRoot MTA, Biodentine, TheraCal LC, and Dycal based on an ARS assay and ALP activity. Compared to Biodentine, ProRoot MTA showed greater osteogenic differentiation ability when tested on hDPSCs (Figure 5 and Figure 6). Similar results have been reported in prior studies. Compared to ProRoot MTA, Biodentine-conditioned media were associated with reduced cellular mineralization according to ALP activity [32,33]. At the molecular level, the Biodentine group showed down-regulated expression of osteogenic markers in differentiated cells [32]. The decreased osteogenic potential of Biodentine may be related to differences in its dissolution rate in osteogenic medium [33]. Our analysis of different Biodentine methods revealed more calcium nodule formation at 21 days when Biodentine was mixed with ddH_2_O rather than with the supplied liquid including CaCl_2_. In contrast, the calcium nodule formation at 28 days did not differ between the Biodentine disk mixed with ddH_2_O versus CaCl_2_ (Figure 7). These results indicated that mineralization activity takes longer to emerge when the Biodentine disk is mixed with the supplied liquid including CaCl_2_.

In our present study, the Biodentine group showed lower calcified nodule formation compared to the TheraCal LC group (Figure 5 and Figure 6). In contrast, previous studies have reported significantly greater calcium nodule formation with Biodentine compared to TheraCal LC [17,29]. Biodentine reportedly shows mineralization efficacy similar to that of MTA in direct capping procedures, and can be considered as a suitable alternative to MTA [18,34]. Light microscopic analysis has confirmed complete dentinal bridge formation directly underneath both Biodentine and ProRoot MTA [34]. Both groups showed layers of well-arranged odontoblasts and odontoblast-like cells, as well as no evidence of inflammatory pulp response below the dentinal bridge [34]. In contrast, discontinuous dentinal bridge formation has been demonstrated in cases of direct pulp capping with TheraCal LC [18]. During direct pulp capping procedures, the non-polymerized resin components (e.g., HEMA, BisGMA, TEGDMA, and UDMA) may come in contact with pulp tissue. Thus, the formation of dispersed mineralization may occur due to non-polymerized monomers discharged from TheraCal LC [18]. Another concern raised about TheraCal CL is the possibility of increased microleakage due to incomplete bridge formation. In our present study, the TheraCal LC group exhibited increasing ALP activity for 7 days, which decreased thereafter, whereas the Biodentine group showed a gradual increase of ALP activity until 14 days (Figure 4). This reflects that Biodentine may take a longer time to reveal osteogenic potential compared to TheraCal LC. The ALP enzyme is the most commonly used early marker of new mineralized tissue formation; therefore, Biodentine may be considered to have beneficial effects on osteogenic potential.

TheraCal LC was reported to release higher levels of Ca^2+^ and OH^−^ compared to ProRoot MTA and Dycal [16]. However, a recent analysis of the hydration reactions of Biodentine and TheraCal LC found that TheraCal LC hydration did not yield calcium hydroxide [27,35]. Gandolfi et al. reported that the hydrophilic resin in TheraCal formulation might permit absorption of some water, and demonstrated that initiating the hydration reaction of the Portland cement particles yielded formation of portlandite or calcium hydroxide [16]. However, Camilleri insisted that although TheraCal LC discharges calcium ions in solution, these calcium ions are not in the hydroxide form [27]. TheraCal LC is a resin-modified calcium silicate cement, and water is not used during the mixing procedure; therefore, the hydration reaction depends on fluid uptake from the surrounding environment [35]. Thus, during the direct pulp capping procedure, if the pulp exposure area does not provide enough water for material hydration, fewer calcium ions may leach from TheraCal LC compared to Biodentine [35]. However, the hydration reaction of Biodentine yields the formation of calcium silicate hydrate and calcium hydroxide [27]. Further research is needed into the chemical hydration properties of TheraCal LC.

One limitation of this study was that we did not consider the three-dimensional culture of hDPSCs. We used experimental disks placed on an insert to reproduce the in vivo pulp capping conditions under which the substances discharged from cements may influence hDPSCs. Previous studies have reported that three-dimensional stem cell spheroids better reproduce clinical conditions compared to a two-dimensional monolayer system [36], and may enhance the survival and therapeutic ability of mesenchymal stem cells [37]. Further studies are needed using a three-dimensional cell culture. Another limitation of this study was that we used set pulp capping material disks. The early effects may differ between materials that have been freshly mixed versus those in their late set state.

## 5. Conclusions

Our present results indicated that ProRoot MTA and Biodentine resulted in higher cell viability and faster cell migration rates compared to Dycal. TheraCal LC showed a lower cell viability and slower cell migration rate compared to ProRoot MTA and Biodentine. The ProRoot MTA and TheraCal LC groups exhibited higher calcium nodule formation on day 21 compared to Biodentine and Dycal, whereas the Biodentine group showed the highest ALP activity on days 10 and 14. Overall, our findings suggest that ProRoot MTA, Biodentine, and TheraCal LC can be used as alternative pulp capping materials, as they showed better results compared to Dycal. Further in vivo clinical research is needed.

## Figures and Tables

**Table 1:** Manufacturers and chemical compositions of various direct pulp capping materials.

Product	Manufacturer	Composition	Batch Number
ProRoot MTA	Dentsply Tulsa Dental Specialties, Tulsa, OK, USA	Portland cement (tricalcium silicate, dicalcium silicate, and tricalcium aluminate), calcium sulfate dihydrate (gypsum), and bismuth oxide	0000186484
Biodentine	Septodont, Saint-Maur-des-Fossés, France	Tricalcium silicate, dicalcium silicate, calcium carbonate, calcium oxide, and zirconium oxide in its powder form Water, calcium chloride, and soluble polymer as an aqueous liquid	B24553
TheraCal LC	Bisco, Schamberg, IL, USA,	Portland cement (calcium silicates), fumed silica, Bis-GMA, and polyglycol dimethacrylate	1900004558
Dycal	Dentsply Caulk, Midford, DE, USA	Base paste: calcium phosphate, calcium tungstate, zinc oxide, iron oxide pigments, and 1,3-butylene glycol disalicylate Catalyst paste: calcium hydroxide, zinc oxide, zinc stearate, titanium oxide, iron oxide pigments, and N-ethyl-o/p-toluene sulphonamide	160801
